# Disseminated *Mycobacterium thermoresistibile* Infection presented with Lymphadenectasis in an AIDS patient: case report and review of literature

**DOI:** 10.1186/s12879-023-08785-w

**Published:** 2023-11-07

**Authors:** Lele Yu, Hu Wan, Jinchuan Shi, Binhai Zhang, Mengyan Wang

**Affiliations:** Department II of Infectious Diseases, Xixi Hospital of Hangzhou, Hangzhou Sixth People’s Hospital, Hangzhou, China

**Keywords:** *Mycobacterium thermoresistibile*, HIV, Case report, NGS, NTM

## Abstract

**Background:**

Nontuberculous mycobacteria disease is a common invasive infectious disease in patients with HIV. However, *Mycobacterium thermoresistibile* association with lymphadenectasis is unusual in AIDS patients.

**Case Presentation:**

This report covers the case of a 25-year-old male AIDS patient infected with *Mycobacterium thermoresistibile*. The case was identified via pathogen-targeted next-generation sequencing (ptNGS).

**Conclusion:**

This is the first report of disseminated *M. thermoresistibile* infection presented with lymphadenectasis in an AIDS patient. Prompt diagnosis and antimicrobial treatment are crucial.

## Introduction

Nontuberculous mycobacteria (NTM) refer to a general term for a large group of mycobacteria except Mycobacterium tuberculosis complex (including Mycobacterium tuberculosis, bovis, African, vole, goat, pinnipedii, suricattae, and mungi) and Mycobacterium leprae. NTM was formerly known as atypical mycobacteria, atypical acid-fast bacilli, etc. More than 190 NTM species and 14 subspecies have been identified, and only a few are pathogenic to humans, which belong to opportunistic pathogens [1]. In recent years, NTM disease has increased rapidly and become an important public health problem threatening human health [2]. In this report, we present a case of *Mycobacterium thermoresistibile* infection lymphadenectasis in an AIDS patient. We also review the clinical characteristics to enhance clinical understanding.

## Case presentation

**Fig. 1:**
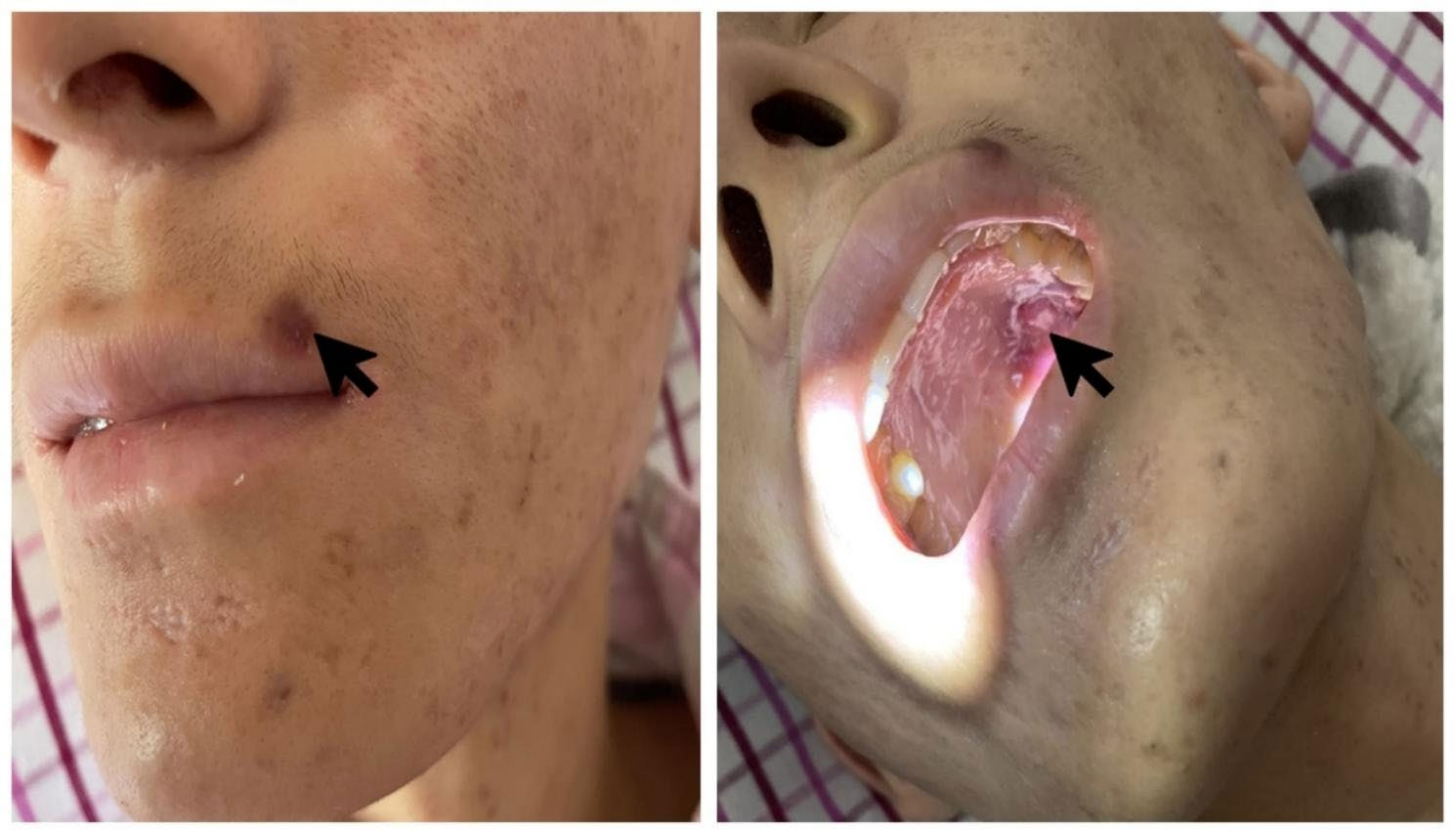
Ulcerative nodules on the patient’s roof of the mouth, and black nodules in corners of the mouth

**Fig. 2:**
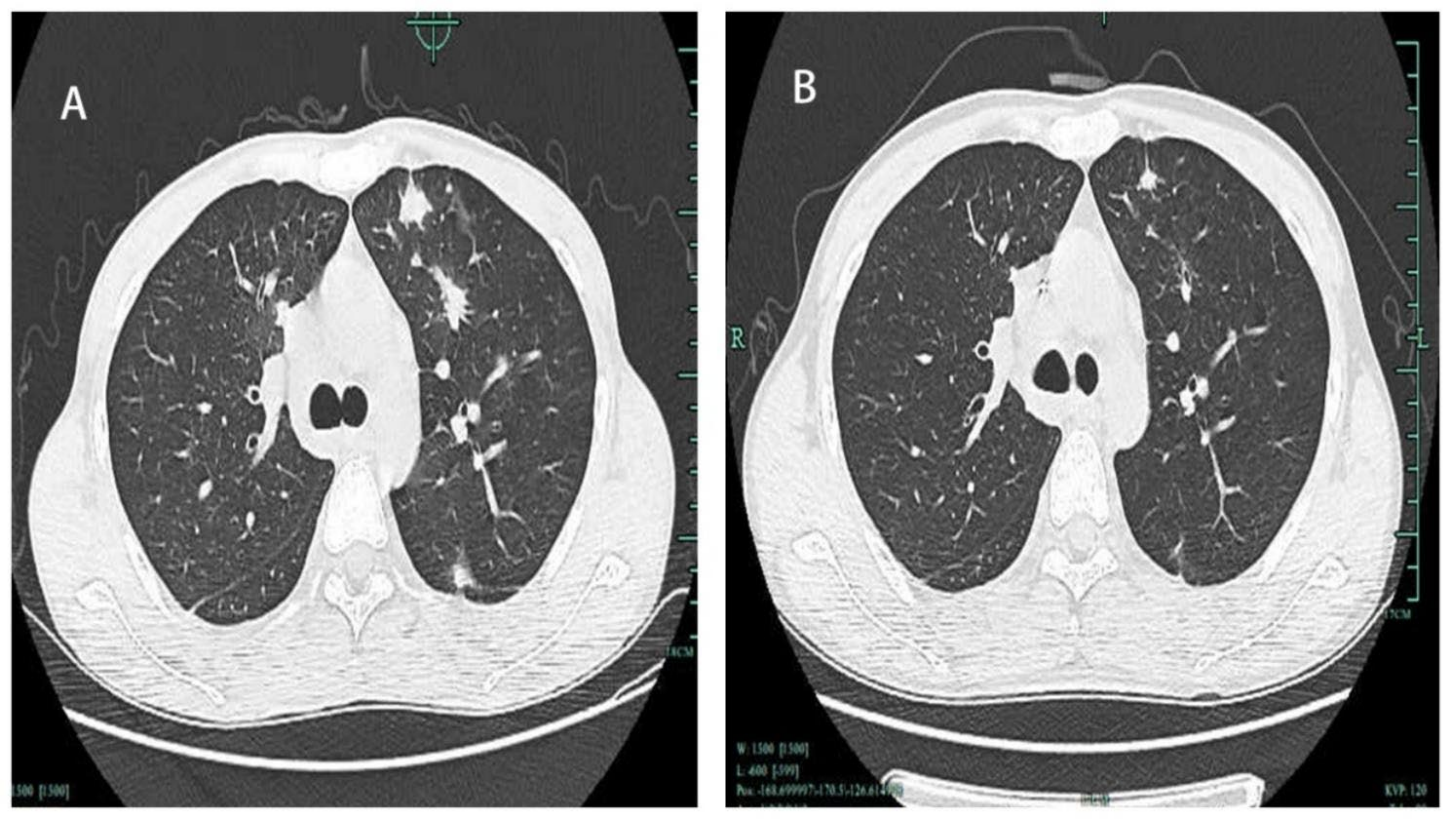
Computed Tomography (CT) of the lungs showed many small patches, nodules, and patchy high-density shadows, with blurred boundaries and uneven density (**A**). After six months of treatment, CT of lung condition improved (**B**)

A 25-year-old male presented with one months of intermittent fever, productive cough, and nine days of black stool. After admission, the patient received active fluid replenishment, blood transfusion, and other supportive treatments. Ulcerative nodules can be seen on the patient’s roof of the mouth, and black nodules can be seen in the eyes and corners of the mouth (Fig. 1), which gradually increased in the past six months. The patient’s physical exam was notable for a temperature of 39.5 °C, heart rate of 115 beats per minute, respiratory rate of 22 breaths per minute, blood pressure of 87/44 mmHg. Neck lymph nodes, axillary lymph nodes, and inguinal lymph nodes were enlargement; low breath sounded in both lungs, no wet and dry rales; unison heartbeat, no pathological murmur; soft abdomen, no tenderness and rebound tenderness. Laboratory findings showed a significant elevation of anti-HIV antibody positive, and the patient’s CD4 T count was 2 cells/ml, HIV-RNA was 6.81 × 10^5IU/ml. Computed Tomography (CT) of the lungs showed many small patches, nodules, and patchy high-density shadows, with blurred boundaries and uneven density (Fig. 2A). One week after admission to the hospital, the patient’s orbital skin biopsy immunohistochemical showed CD34(+) CDX2(-) D2-40(+) HHV8(+) Ki-67(50%+), which was taken to suggest Kaposi’s sarcoma. According to guidelines, the patient received Liposomal Doxorubicin (20 mg/m2 IV every three weeks) for Kaposi’s sarcoma.

However, the patient was still running a high fever, and sputum was sent for microscopy and culture, the Xpert MTB/RIF assay was negative, but acid-fast staining of blood was positive after 24 days since admission. The interferon-gamma release assay (IGRA) of tuberculosis (TB) in blood was positive. Four days after those positive results, Pathogen-targeted next-generation sequencing (ptNGS, a multiple PCR-based targeted NGS technique) of cervical lymph node tissue reported *Mycobacterium thermoresistibile (M. thermoresistibile)*. Results of ptNGS reported 1 × 10^6^ unique reads of *Mycobacterium thermoresistibile*, and there were no resistance mutations at the loci. After reviewing the drug-sensitivity panel of the organism, the decision was made to start the patient on Isoniazid (0.3 g q.d.), ethambutol (0.75 g q.d.), rifabutin (0.3 g q.d.), azithromycin (0.5 g q.d.) and Levofloxacin (0.5 g q.d.). The duration of treatment is expected to be at least one year, determined by the CD4 + T count.

After six months of treatment, the patient’s symptoms were alleviated, enlarged lymph nodes shrank, the temperature returned to normal, lung condition improved slowly (Fig. 2B), the count of CD4 + T cells increased gradually, and the HIV load dropped greatly.

## Discussion

NTM disease refers to human infection with NTM, which causes lesions in related tissues and organs. *Mycobacterium thermoresistibile* is a rapid-growth form of NTM. Japanese scholars Tsuk first isolated the bacteria from the soil, then it was isolated in respiratory tract of infected animals in 1966 [3]. It was previously thought to be non-pathogenic to humans, but in 1981, Weitzman [4] reported the first human case of pneumonia caused by thermostable mycobacteria. *Mycobacterium thermoresistibile* can cause both intrapulmonary and extrapulmonary disease. In our literature review, eight cases of *M. thermoresistibile* infection have been reported (including the current case) (Table 1). The main clinical manifestations were cough (dry cough or phlegm), absence of systemic symptoms or low-grade fever, fatigue, and weight loss. Patients with a chronic course of the disease may be associated with immunodeficiency. Chest imaging showed inflammatory lesions and single or multiple cavities or only multiple nodules. Severe cases showed diffuse patchy shadows in both lungs. Liu et al. reported a second pulmonary *M. thermoresistibile* infection in an immunocompromised host with hypogammaglobulinaemia [5]. Extrapulmonary lesions due to *Mycobacterium thermoresistibile* had cutaneous infection. Generally, there was a history of trauma, manifested as local abscess formation and delayed healing. A diabetic patient who underwent a heart transplant had an infection near the surgical scar in which *Mycobacterium thermoresistibile* was detected [6]. Wolfe et al. reported on the formation of breast abscesses by *M. thermoresistibile* following augmentation mammaplasty in 1992 [7]. Whereafter, a female patient with a 6-month history of a violaceous indurated plaque that developed after trauma was reported in 2000 [8]. LaBombadi et al. [9] reported on a *M. thermoresistibile* infection following knee-replacement surgery. In 2009, Neonakis et al. [10] reported on the isolation of M. thermoresistibile from a sputum culture of a patient from the island of Crete, Greece, with chronic obstructive pulmonary disease (COPD), diabetes and purpura, which was the first report of M. thermoresistibile isolation from a clinical sample in Europe. In conclusion, lymphadenitis has not been reported. To our knowledge, this is the first report of disseminated *M. thermoresistibile* infection presented with lymphadenectasis in an AIDS patient. Infections with NTM belong to the AIDS-defining illnesses of HIV infection. Severe immunosuppression with CD4 + lymphocyte counts lower than 50 cells/microl was a risk factor for the acquaintance of NTM infections [11]. Disseminated NTM disease was rare in individuals with immune defects [12]. The results of susceptibility testing were not uniform in vitro. Weitzman et al. [4] found that the strain was sensitive to ethambutol (ETH) (5 and 10 mg/L), rifampicin (RMP) (1 mg/L), high-concentration streptomycin (STR)(10 mg/L), resistant to low-concentration STR (2 mg/L), isoniazid (0.5,1 and 5 mg/L) and para-amino-salicylic acid (PAS) (2 mg/L). Liu et al. [5] reported that susceptibility testing of M. thermoresistibile showed sensitivity to RMP, ETH, STR, kanamycin and resistance to isoniazid (INH) and PAS. Neeley et al. [6] noticed that the response to the drugs given (RMP, ETH and INH) was slow. Wolfe et al. [7] reported the result of antimicrobial test showed susceptibility to amikacin (AN), ciprofloxacin (CIP), doxycycline (DOX), RMP, ETH, STR, capreomycin, tetracycline and resistance to ETN, PAS, INH, and ofloxacin (OFL). The isolation of M. thermoresistibile was colonization considered by Neonakis et al. [10] and AN, cefoxitin (FOX), CIP, clarithromycin (CLA), DOX, imipenem (IMP), and trimethoprim–sulfomethoxazol (SXT) to which was susceptible.

**Table 1:** Demographics, Underlying conditions, Clinical Features, Treatment, and Outcomes of Patients with *M. thermoresistibile* infection

Author (Year)	Age/sex	Underlying conditions	Presenting Symptoms	How Was Diagnosis Made?	Site	Treatment (Duration)	Outcome
Weitzman (1981)	middle-aged/F	NR	cough, fever	Sputum culture	respiratory tract	RMP, ETH and STR (NR)	improve
Liu (1984)	64/M	hypogammaglobulinaemia	cough and purulent nasal drainage	tissue of lung biopsy	lung	RMP, ETH and STR (4 weeks)	improve
Neeley (1989)	41/M	Diabetes, cardiac transplantation	cutaneous lesion	purulent material culture	skin	RMP, ETH and INH (3 months)	slow response
Wolfe (1992)	41/F	augmentation mammaplasty	breast abscesses	purulent material culture	breast skin	RMP, ETH and INH (16 months)	completely resolved
Cummings (2000)	NR/F	A coinfection with M. fortuitum	cutaneous lesion	tissue samples culture	skin	levofloxacin and DOX (3 months)	completely resolved
LaBombardi (2005)	73/F	knee-replacement surgery	swelling in the left knee and fevers	tissue samples culture	skin	MOX and linezolid (later replaced by DOX) (7 months)	improve
Neonakis (2009)	67/M	chronic obstructive pulmonary disease (COPD), diabetes and purpura	fever, cough, dyspnea, weakness and acute purpura	Sputum culture	lung	Ciprofloxacin (NR)	improve
Our case	25/M	HIV, Kaposi’s sarcoma	Multiple systemic lymph node enlargement, fever	Biopsy specimens of lymph nodes ptNGS	lymph node	INH, ETH, RMP, azithromycin and Levofloxacin	improve

rifampicin (RMP), ethambutol (ETH), streptomycin (STR), isoniazid (INH), para-amino-salicylic acid (PAS), Moxifloxacin (MOX), doxycycline (DOX), Pathogen-targeted next-generation sequencing (ptNGS), not report (NR)

*M. thermoresistibile* can grow at 37 ~ 45 °C, and the most suitable growth temperature is 42 °C, which is considered to be a unique population between slow and fast growing bacteria [4]. Because of the atypical growth rate and colonies formed below 42 °C, it is possible to misdiagnose other NTMs such as *Mycobacterium gordonae* [8]. Sometimes, it was difficult to achieve clearcut identification from culture, and we used molecular techniques to identify uncommon mycobacteria in this case. Previous studies have proven that ptNGS has a number of advantages of sensitivity, timeliness, and economy over mNGS. The ptNGS was developed to identify pathogens in respiratory tract infection or mycobacterium infection cases, the details of ptNGS were described previously [13]. It is known that excessive consumption of sequencing resources by human-derived nucleic acids in mNGS. Therefore, it seems that ptNGS has the advantages of detection sensitivity not affected by human genome, background bacteria, pathogen genome size. And ptNGS has lower detection cost, reduced sample transportation requirements, and quantitative detection of pathogens [14]. Here, we considered the NTM infection in this case after positive of acid-fast staining. And ptNGS technology was used for the molecular identification of NTM.

There were seven cases of *M. thermoresistibile* infection up to now, in view of the special growth conditions of the bacteria, the possibility of misdiagnosis or missed diagnosis in previous NTM cases cannot be ruled out. Due to *M. thermoresistibile* infection can occur in both healthy and immunocompromised people, clinicians or bacterial examiners should be alert to this pathogen. Traditional acid-fast staining does not distinguish between NTM and Mycobacterium tuberculosis, and culture is time-consuming and sometimes shows false-negative results. NGS can successfully identify infectious pathogens of unknown origin in samples like blood, bone marrow, and gastrointestinal tract. Compared with culture-based methods, NGS offers significant advantages in terms of high detection efficiency and speed. The detection accuracy and positive rate of microorganisms with NGS are higher.

## Data Availability

The datasets used and/or analysed during the current study available from the corresponding author on reasonable request.
